# Effects of Telerehabilitation Interventions on Heart Failure Management (2015-2020): Scoping Review

**DOI:** 10.2196/29714

**Published:** 2021-11-01

**Authors:** Cathrine Skov Schacksen, Nanna Celina Henneberg, Janusiya Anajan Muthulingam, Yuh Morimoto, Ryuichi Sawa, Masakazu Saitoh, Tomoyuki Morisawa, Nobuyuki Kagiyama, Tetsuya Takahashi, Takatoshi Kasai, Hiroyuki Daida, Jens Refsgaard, Malene Hollingdal, Birthe Dinesen

**Affiliations:** 1 Laboratory for Welfare Technology - Telehealth & Telerehabilitation, Sport Sciences - Performance and Technology Department of Health Science and Technology Aalborg University Aalborg East Denmark; 2 Department of Clinical Medicine Aalborg University Aalborg Denmark; 3 Mech-Sense Department of Radiology Aalborg University Hospital Aalborg Denmark; 4 Faculty of Health Science Juntendo University Tokyo Japan; 5 Department of Physical Therapy Faculty of Health Science Juntendo University Tokyo Japan; 6 Department of Digital Health and Telemedicine R&D Faculty of Health Science Juntendo University Tokyo Japan; 7 Department of Cardiovascular Biology and Medicine Juntendo University Graduate School of Medicine Tokyo Japan; 8 Department of Cardiology Regional Hospital Viborg Viborg Denmark

**Keywords:** heart failure, telerehabilitation, quality of life, physical capacity, depression, anxiety, telehealth, rehabilitation, cardiac rehabilitation, cardiovascular disease, CVD, mental health, adherence, quality of life, physical capacity

## Abstract

**Background:**

Heart failure is one of the world’s most frequently diagnosed cardiovascular diseases. An important element of heart failure management is cardiac rehabilitation, the goal of which is to improve patients’ recovery, functional capacity, psychosocial well-being, and health-related quality of life. Patients in cardiac rehabilitation may lack sufficient motivation or may feel that the rehabilitation process does not meet their individual needs. One solution to these challenges is the use of telerehabilitation. Although telerehabilitation has been available for several years, it has only recently begun to be utilized in heart failure studies. Especially within the past 5 years, we now have several studies focusing on the effectiveness of telerehabilitation for heart failure management, all with varying results. Based on a review of these studies, this paper offers an assessment of the effectiveness of telerehabilitation as applied to heart failure management.

**Objective:**

The aim of this scoping review was to assess the effects of telerehabilitation in the management of heart failure by systematically reviewing the available scientific literature within the period from January 1, 2015, to December 31, 2020.

**Methods:**

The literature search was carried out using PubMed and EMBASE. After duplicates were removed, 77 articles were screened and 12 articles were subsequently reviewed. The review followed the PRISMA-ScR (Preferred Reporting Items for Systematic Reviews and Meta-Analyses for scoping reviews) guidelines. As measures of the effectiveness of telerehabilitation, the following outcomes were used: patients’ quality of life, physical capacity, depression or anxiety, and adherence to the intervention.

**Results:**

A total of 12 articles were included in this review. In reviewing the effects of telerehabilitation for patients with heart failure, it was found that 4 out of 6 randomized controlled trials (RCTs), a single prospective study, and 4 out of 5 reviews reported increased quality of life for patients. For physical capacity, 4 RCTs and 3 systematic reviews revealed increased physical capacity. Depression or depressive symptoms were reported as being reduced in 1 of the 6 RCTs and in 2 of the 5 reviews. Anxiety or anxiety-related symptoms were reported as reduced in only 1 review. High adherence to the telerehabilitation program was reported in 4 RCTs and 4 reviews. It should be mentioned that some of the reviewed articles described the same studies although they employed different outcome measures.

**Conclusions:**

It was found that there is a tendency toward improvement in patients’ quality of life and physical capacity when telerehabilitation was used in heart failure management. The outcome measures of depression, anxiety, and adherence to the intervention were found to be positive. Additional research is needed to determine more precise and robust effects of telerehabilitation.

## Introduction

Heart failure (HF) is one of the world’s most frequently diagnosed cardiovascular diseases [1]. It is estimated that throughout the world, more than 37 million people have been diagnosed with HF [1]. HF is highly prevalent, and it is predicted that the prevalence will increase in the future and will therefore be a growing burden on the health sector [2]. Cardiac rehabilitation is an important part of HF management, as it aims to improve patients’ recovery and enhance their functional capacity, psychosocial well-being, and health-related quality of life (QoL). Cardiac rehabilitation focuses on weight control, psychosocial coping, disease management, and improving both physical activity and diet management [3]. Cardiac rehabilitation has often been conducted in a rehabilitation facility, which can be a challenge when patients have limited means of transportation. Patients in cardiac rehabilitation may also lack motivation, especially if they feel that the rehabilitation process is not adequately tailored to their needs [3,4]. A strategy for managing these challenges is the use of telerehabilitation. Telerehabilitation is defined as the delivery of rehabilitation services via information and communication technologies and generally takes place in the patient’s home [4-6].

A review from 2015 assessed whether telerehabilitation was effective for improving physical or functional outcomes in patients with cardiopulmonary diseases [7]. This review found only 4 studies carried out between 2000 and 2012 that had utilized telerehabilitation to manage HF [8-11]. The use of telerehabilitation for assisting patients with HF has thus been available for several years. However, it was not used as a part of rehabilitation for HF management among patients in many studies until a few years ago. In recent years, evidence assessing the effectiveness of telerehabilitation and HF management has grown, showing varying results. Nevertheless, there remains a lack of reviews on the general effectiveness of telerehabilitation for HF management. The aim of this review was to investigate the effects of telerehabilitation in the management of HF by systematically reviewing the most recent, available scientific literature from the 6-year period from January 1, 2015, to December 31, 2020.

## Methods

### Search Strategy

A research protocol for reviewing the available literature was designed. The protocol included inclusion and exclusion criteria, type of studies, identification of telerehabilitation technologies, intervention duration, and outcome measurements. We conducted a literature search of randomized controlled trials (RCTs), prospective intervention studies, reviews, and meta-analyses, all of which aimed to examine the effectiveness of telerehabilitation on patients with HF. Inclusion and exclusion criteria are summarized in Textbox 1. The literature search was performed using the PubMed and Excerpta Medica (EMBASE) databases, as these two databases were thought to contain many telerehabilitation studies. In searching through the two databases, the following search words were used in combinations: “heart failure,” “HF,” “telecardiology,” and “telerehabilitation.” MeSH (Medical Subject Headings) terms were used, where possible, on PubMed, and Emtree terms were used, where possible, on EMBASE. The literature search was limited to the time frame January 1, 2015, to December 31, 2020. Multimedia Appendix 1 provides the protocol with the full search strategy.

**Inclusion and exclusion criteria for the search.**

**Inclusion criteria**

- A home telerehabilitation program for patients with heart failure (HF)
- A comparative study with telerehabilitation and traditional home care or other approaches
- Participants who are older than 18 years
- At least one of the following outcome measures were reported to have been used: quality of life, physical capacity, depression or anxiety, or adherence to the intervention
- Randomized controlled trials, prospective intervention studies, reviews, and meta-analyses published in the preceding 6 years (January 1, 2015, to December 31, 2020)

**Exclusion criteria**

- Languages other than English
- Studies where results from patients with chronic diseases other than HF are not reported separately from the results pertaining to patients with HF
- Protocols
- Only abstracts

### Outcome Measures

In assessing the effectiveness of telerehabilitation, the various studies used a range of outcome measures. The most common effect measures in HF and telerehabilitation are QoL, physical capacity, depression or anxiety, and adherence to the intervention [2]. Therefore, our review used these four outcomes as well.

### Screening

**Figure 1:**
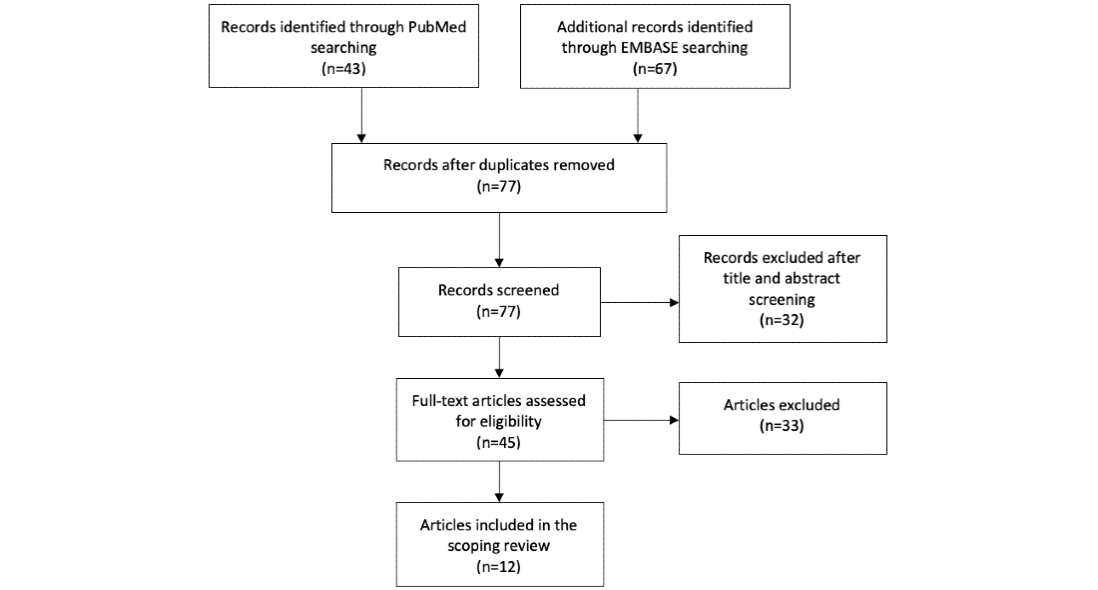
Flow diagram of the screening and selection process of articles.

The review has followed the PRISMA-ScR (Preferred Reporting Items for Systematic Reviews and Meta-Analyses for scoping reviews) guidelines [12]. First, 2 authors (CSS and NCH) independently performed the literature retrieval. Then, 2 authors (CSS and NCH) independently screened the abstracts identified during the search. Based on the screening of the titles and content of the abstract, each article was assessed as to whether it fulfilled the inclusion or exclusion criteria. Articles that were deemed relevant and met the inclusion criteria were selected for inclusion in our study. Disagreements between the 2 reviewers were resolved by discussion until consensus was reached. The initial systematic literature search resulted in 110 articles on HF. The final selection of relevant articles comprised 12 articles on HF and telerehabilitation [7,13-23]. Figure 1 outlines the screening process.

### Data Extraction

The following information was collected from all included studies: references, design of the study, sample size, severity of HF (reported per the New York Heart Association Functional Classification), intervention type, technology, duration of intervention and follow-up, health care utilization, outcomes (QoL, physical capacity, depression or anxiety, adherence to the intervention), and questionnaires or tests used to measure QoL, physical capacity, and depression or anxiety.

### Synthesis of Results

The synthesis of the review was performed in 3 steps:

1. For HF and telerehabilitation, an overview of the main results of the studies that are relevant to this review were presented in a tabular format.
2. Only significant results were reported in the overviews. Hence, we did not report tendencies.
3. A summary of HF and telerehabilitation within the time frame 2015-2020 was presented in a tabular format.

## Results

### HF and Telerehabilitation

For HF, a total of 6 RCTs [13,15,17,20,22,23], 1 prospective study [16], and 5 reviews [7,14,18,19,21] were included. The sample size in the RCTs varied from 17 to 850 patients with HF. The telerehabilitation interventions described in the studies varied from 8 weeks to 5 months, and follow-up periods varied from 12 weeks to 16 months. The technologies discussed in the studies were educational interventions, telephone monitoring, exercise programs, teleconsultations, video-based consultations, mobile phones, and forwarding technologies.

### Study Selection, Characteristics, and Outcomes

Table 1 provides an overview of the studies on telerehabilitation and HF published in the 2015-2020 period. The outcome measures were QoL, physical capacity, depression or anxiety, and adherence to the intervention, and these are presented in Table 2. The boxes containing the abbreviation “N/A” (not applicable) indicate that the parameter was evaluated in the specific study but that the result of the evaluated parameter was not significant. An arrow indicates that the result of the specific outcome measure was significantly different, which could either be upward, meaning that the outcome measure was increased during the intervention, or downward, meaning that the outcome measure was decreased. The arrow is followed by either “the intervention group” or “in both groups,” which indicates the group(s) to which the study results relates. Furthermore, if the questionnaires or tests used to measure the outcome measures were reported in the study, the results of the outcomes are followed by the name of the questionnaires or tests in brackets. For the review articles, the number of studies showing significant results of the specific outcome are listed in brackets. The control group category refers to patients with HF receiving conventional rehabilitation care, where some received education with no exercise and others received center-based rehabilitation with exercise. The outcome adherence is reported in Table 2 as reported in the studies. “Adherence” refers to the degree to which participants in the study follow the intervention assigned to them; thus, high adherence or a high percentage of adherents refers to those participants showing behavior that corresponds with the intervention.

**Table 1:** Overview and characteristics of studies on telerehabilitation and heart failure (HF).

Reference	Country	Type of study and sample size	Severity of HF (NYHA^a^ Functional Classification)	Intervention	Technology	Duration of intervention and follow-up
Brunetti et al [18]^b^	Italy	Expert review	—^c^	Home-based telerehabilitation; telemanagement and home-based telesurveillance, remote patient monitoring; home-based nurse telemanagement by telephone and interactive teleconsultation; interactive telecommunication; cooperation between general practitioners and a telemonitoring HF clinic	Phone calls, videoconferencing, ECG^d^ recorder with transmission	—
Hamilton et al [14]	Australia	Systematic review (4 HF studies)	II, III, and IV	Mobile health home-based telerehabilitation	Smartphone with apps, digital weight scale, automatic blood pressure monitor, and ECG recorder; all transmitted via Bluetooth	—
Hwang et al [7]	Australia	Systematic review (4 HF RCTs^e^)	—	Home-based telerehabilitation	Telephone communication, ECG recorder	8-36 weeks of intervention and follow-up from none to 1 year
Hwang et al [23]	Australia	RCT (N=53; intervention group, n=24; control group, n=29)	I, II, and III	Group home-based telerehabilitation with real-time online video conferencing exercise and education	Computer, video conferencing program, electronic slide presentations with educational topics, telephone contact, automatic sphygmomanometer, finger pulse oximeter	12 weeks and follow-up after 12 weeks
Nakayama et al [16]	Japan	Prospective intervention study (N=236; home-based intervention group, n=30; outpatient-based intervention group, n=69; control group, n=137)	IV	Home-based telerehabilitation, and outpatient-based telerehabilitation with telephone consultations	Telephone communication and consultations, pedometer, educational rehabilitation DVD with exercises, blood pressure, weight scale	30 days
Peng et al [15]	China	RCT (N=98; intervention group, n=49; control group, n=49)	I, II, and III	Home-based telerehabilitation with a telehealth exercise training program	Text-based, audio, or video conversations regarding follow-up; computer; and a home-based platform (WeChat) for communicating with nurses	8 weeks and follow-up after 4 months
Piotrowicz et al [13]	Poland	RCT (N=111; intervention group, n=77; control group, n=34)	II and III	Home-based telemonitored walking training	ECG recorder (EHO-MINI device), blood pressure monitor, weight scale; all data were transmitted via a mobile phone to the monitoring center	8 weeks
Piotrowicz et al [20]	Poland	RCT (N=131; intervention group, n=77; control group, n=75)	II and III	Home-based telemonitored walking training	ECG recorder (EHO-MINI device); transmitted via a mobile phone to the monitoring center	8 weeks
Piotrowicz et al [21]^b^	Poland	Review (6 HF studies)	—	Home-based telerehabilitation	Telephone communication or transmission of data via telephone, ECG recorder, blood pressure monitor, weight scale, saturation, respiration, cardiovascular implantable electronic device, hemodynamic implantable electronic devices	—
Piotrowicz et al [17]	Poland	RCT (N=111; intervention group, n=77; control group, n=34)	II and III	Home-based telemonitored walking training	ECG recorder (EHO-MINI device), blood pressure monitor, weight scale; all data were transmitted via a mobile phone to the monitoring center	8 weeks
Piotrowicz [19]	Poland	Expert review (5 studies)	II and III	Home-based telerehabilitation	Telesupervised exercise training	—
Piotrowicz et al [22]	Poland	RCT (N=850; intervention group, n=425; control group, n=425)	I, II, and III	Hybrid home-based telerehabilitation with remote monitoring of training at patients’ homes	ECG recorder (EHO-MINI device), blood pressure monitor, weight scale; all data were transmitted via a mobile phone to the monitoring center	9 weeks and follow-up over a period of 14-16 months

^a^NYHA: New York Heart Association.

^b^Results pertaining to patients with heart failure were difficult to distinguish from those of patients with other cardiac diseases.

^c^Parameter not evaluated.

^d^ECG: echocardiogram.

^e^RCT: randomized controlled trial.

**Table 2:** Overview of outcomes of studies on telerehabilitation and heart failure.

Reference	Quality of life	Physical capacity	Depression or anxiety	Adherence to the intervention
Brunetti et al [18]^a^	↑ (1 study)	↑ (1 study)	—^b^	—
Hamilton et al [14]	↑ (SF-36^c^, 1 study)	—	—	High adherence in all studies included; reported as 94% and 95% in 2 of the studies
Hwang et al [7]	↑ (3 studies)	↑ (6MWT^d^, 1 study) in the intervention group	—	Higher adherence in telerehabilitation than center-based exercise (2 studies)
Hwang et al [23]	↑ (EQ-5D^e^) in both groups	N/A ^f^ (6MWT, 10MWT^g^)	—	71% adherence in the intervention group
Nakayama et al [16]	↑ (EQ-5D) in the intervention group	—	—	—
Peng et al [15]	↑ (MLHFQ^h^) in the intervention group	↑ (6MWT) in the intervention group	N/A (HADS^i^) for both depression and anxiety	—
Piotrowicz et al [13]	↑ (SF-36) in the intervention group	↑ (peak oxygen consumption [VO_2_], 6MWT) in the intervention group	—	94.7% adherence in the intervention group
Piotrowicz et al [20]	↑ (SF-36) in both groups	↑ (physical capacity subscale in SF-36) in both groups	—	—
Piotrowicz et al [21]^a^	↑ (2 studies)	↑ (2 studies)	↓ depression (1 study) and ↓ anxiety (1 study)	Higher adherence to telerehabilitation than usual care
Piotrowicz et al [17]	—	↑ (cardiopulmonary exercise test peak VO_2_) in the intervention group	↓ (BDI^j^) depression in both groups	—
Piotrowicz [19]	↑ in both groups	↑ (3 studies)	↓ depression (1 study)	High adherence in the intervention group (2 RCTs^k^)
Piotrowicz et al [22]	N/A (SF-36); significant differences were reported only between groups and not internally within groups	N/A (6MWT, cardiopulmonary exercise test, peak oxygen consumption peak VO_2_); significant differences were reported only between groups and not internally within groups	—	88.4% adherence in the intervention group

^a^Results pertaining to patients with heart failure patients were difficult to distinguish from those of patients with other cardiac diseases.

^b^Parameter not evaluated.

^c^SF-36: 36-Item Short Form Survey.

^d^6MWT: 6-minute walk test.

^e^EQ-5D: European Quality of Life–5 Dimensions.

^f^N/A: not applicable.

^g^10MWT: 10-minute walk test.

^h^MLHFQ: Minnesota “Living with Heart Failure” Questionnaire.

^i^HADS: Hospital Anxiety and Depression Scale.

^j^BDI: Beck Depression Inventory.

^k^RCT: randomized controlled trial.

### Synthesis of Studies on HF and Telerehabilitation

The studies on the use of telerehabilitation for patients with HF indicated that 4 out of 6 RCTs, 1 prospective study, and 4 out of 5 reviews reported an increase in QoL (Table 3). In terms of physical capacity, 4 RCTs and 3 systematic reviews reported increased physical capacity. Depression or depressive symptoms were reported as reduced in 1 RCT and 2 reviews. Anxiety or anxiety-related symptoms were reported as reduced in 1 of 6 reviews. High adherence to the telerehabilitation program was reported in 4 RCTs and 4 reviews. It should be noted that some of the reported articles described the same studies but presented different outcome measures.

**Table 3:** Synthesis of studies on heart failure and telerehabilitation, 2015-2020.

Study type	Quality of life	Physical capacity	Depression or anxiety	Adherence to the intervention
Randomized controlled trial (n=6)	↑ (4 studies)	↑ (4 studies)	↓ depression (1 study)	High (4 studies)
Prospective study (n=1)	↑ (1 study)	—^a^	—	—
Review (n=5)	↑ (4 studies)	↑ (3 studies)	↓ depression (2 studies) and ↓ anxiety (1 study)	High (4 studies)

^a^Parameter not evaluated.

## Discussion

### Principal Findings

The aim of this study was to investigate the effects of telerehabilitation on the management of HF by reviewing the available scientific literature within the period from January 1, 2015, to December 31, 2020. This review was based on the following outcomes: QoL, physical capacity, depression or anxiety, and adherence to the telerehabilitation intervention. Overall, it was found that telerehabilitation had a positive influence on the outcome measures. The 12 articles reviewed here showed wide variation in terms of the numbers of patients included, the duration of the intervention, the duration and absence of a follow-up period, the outcome measurements, and the technologies used.

Telerehabilitation interventions for patients with HF showed that patients who participated in telerehabilitation programs improved their QoL compared to those in conventional rehabilitation programs [7,13-18,20,21,23]. However, 2 RCTs showed a significant improvement in QoL both in the telerehabilitation and the control groups [20,23]. Furthermore, 1 study did not show an improvement in QoL internally in the groups; however, a significant difference in QoL between the groups was seen, with QoL being highest in the telerehabilitation group compared to the control group [22]. These results indicate that in most cases, telerehabilitation helped increase QoL in patients with HF compared to conventional care.

Increased physical capacity was seen in 7 out of the 10 studies that reported this outcome [13,15,17-21]. Physical capacity was measured by using a variety of outcomes in the identified studies, but most studies employed the 6-minute walking test for assessing the effectiveness of telerehabilitation. However, physical capacity or physical activity can be measured by other methods. In a RCT with patients with HF enrolled in a telerehabilitation program, Gade et al [24], whose study was not included in this review, measured physical activity using a Fitbit step counter for 1 year. The study found no increase in the number of steps. However, a significant correlation was found between the increased number of steps and the reduction in the ejection fraction. Furthermore, it was found that a step counter can be a useful tool to help patients monitor their own physical activity [24]. These findings suggest that measurement of patients’ physical activity can be carried out using technologies such as a step counter instead of tests.

All the included studies in our review used home-based telerehabilitation as their intervention. However, there were variations in the way the patients were approached, where some were group-based and others were individually based. Only 1 out of 12 studies were group-based [23], thereby indicating that the home-based intervention is most commonly used as a single-based intervention. Many of these studies used the same types of technologies, such as cell phones, which was seen in 8 of the included studies [7,14,16-18,20-22]. Other types of technologies used were different types of ECG recorders, blood pressure meter, weight scale, saturation device, respirometer video, oximeter sphygmomanometer, computer, and others (Table 1) [7,13-23]. However, other studies on telerehabilitation use among patients with HF and patients with other cardiac disorders utilized technologies such as pedometers, sleep sensors, tablets, online portals, and apps [24-26]. This suggests that other studies will employ additional, more advanced, and newer technologies than the ones mentioned in this review, such as wearables. The implication is that future research will need to focus on the potential of these technologies as part of a telerehabilitation regime for patients with HF.

Depression was used as an outcome measure in only 4 of the 12 studies [15,17,19,21]. Three of these 4 studies showed a significant decrease in depression with telerehabilitation use among patients with HF [17,19,21]. Only 2 of the 4 studies had anxiety as an outcome measure [15,21]. Piotrowicz et al [21] found a decrease in both anxiety and depression when using telerehabilitation. Peng et al [15] investigated the effects of home-based telehealth exercise training on both depression and anxiety. However, the results proved to be not significant, thereby indicating that the home-based telehealth exercise program in this study did not produce any significant improvement in either of the two outcome measures [15]. Other studies reported similar results in patients with cardiac disorders, thus showing that telerehabilitation can have positive effects on depression and anxiety outcome measures [26-28].

Patients’ adherence to the interventions was measured in 7 of the studies included, and all 7 reported high adherence to the telerehabilitation intervention [7,13,14,19,21-23]. Furthermore, in most of the studies, adherence was reported to be higher in the telerehabilitation group compared to the standard care group. Studies reported adherence using different measures like self-reported activity, daily phone contacts, number of exercises per week, or number of rehabilitation sessions attended. As telerehabilitation is often home-based, there may be uncertainty regarding the degree to which patients actually adhere to the program. Daily phone contacts with patients, which was the measure used in 2 studies [13,22], ensured a more stable monitoring of adherence; however, phoning patients every day requires considerable resources. Another, less labor-intensive method of monitoring adherence to a program could be the use of a pedometer, as the patient would be able to monitor their physical activity without any subjective reporting, assuming that the results are then accurately transmitted.

Telerehabilitation is still a developing feature in the management of HF. There is a growing body of literature on the effect of telerehabilitation compared to conventional care in both patients with HF as well as other diseases [6]. This is especially relevant now since the COVID-19 pandemic has drawn attention to and accelerated the use of telerehabilitation, due to restrictions on the use of physical therapy sessions in many countries. Therefore, it is likely that the use of telerehabilitation will become more integrated into clinical practice in the future, as the pandemic has stimulated the use of new approaches for rehabilitation for patients with chronic diseases [29]. This scoping review has been conducted in order to examine to what extent telerehabilitation has been implemented for patients with HF and to document the impact of telerehabilitation for patients with HF in terms of QoL, physical capacity, depression, anxiety, and intervention adherence. The evidence from our review indicates that future research within telerehabilitation for patients with HF should focus on larger-scale clinical trials, longer duration of interventions and follow-up periods, and patients’ adherence to telerehabilitation interventions.

### Limitations

As telerehabilitation is still a relatively new approach to cardiac rehabilitation, studies of the use of telerehabilitation for patients with HF are often not comparable. There is still no consensus on optimal outcomes, which makes it a challenge to compare studies since outcomes are measured differently. Furthermore, reviews often focus on cardiac telerehabilitation concerned with various cardiac diseases, which again makes it a challenge in distinguishing HF results from other cardiac diseases in reviews.

The studies reviewed here did not focus extensively on patient education. Instead, their focus was on physical tests. However, we are aware of telerehabilitation programs where patient education is part of the program [25].

### Conclusion

The review has shown that the effect of telerehabilitation in patients with HF is still relatively new. Our review indicates a relative consensus that use of telerehabilitation for patients with HF helps improve patients’ QoL and their physical capacity. The outcome measures of depression and anxiety were found to be reduced as well. Moreover, high adherence to telerehabilitation interventions was found in most of the studies. However, there is still no consensus on how and which outcomes should be measured within telerehabilitation. Therefore, additional research is needed to determine more precise and robust effects of using telerehabilitation for patients with HF.

## Appendix

Multimedia Appendix 1 Scoping review protocol.

## Abbreviations

HF: heart failure
MeSH: Medical Subject Headings
PRISMA-ScR: Preferred Reporting Items for Systematic Reviews and Meta-Analyses for scoping reviews
QoL: quality of life
RCT: randomized controlled trial
