# Supplementary material for: Effects of Telerehabilitation Interventions on Heart Failure Management (2015-2020): Scoping Review
Source: JMIR Rehabil Assist Technol. 2021 Nov 1;8(4):e29714. doi: 10.2196/29714 (PMC8593801; doi:10.2196/29714)
Supplement: Multimedia Appendix 1 [file rehab_v8i4e29714_app1.docx]

# Protocol

| Review title and timescale |
| --- |
| Review title |
| Effects of Telerehabilitation Interventions in Heart Failure 2015-2020: Scoping Review |
| Start date |
| January 2^nd^ 2021 |
| Completion date |
| March 15^th^ 2021 |
|  |
| Review team details |
| Corresponding author |
| Cathrine Skov Schacksen, BSc. |
| Contact e-mail |
| cass@hst.aau.dk |
| Address |
| Aalborg University, Fredrik Bajers Vej 7, Bld. A1,  9220 Aalborg East Denmark |
| Phone number |
| +4561285904 |
| Organizational affiliation of the review |
| Laboratory for Welfare Technology – Telehealth & Telerehabilitation,  Sport Sciences - Performance and Technology, Department of Health Science and Technology |
| Review team members |
| Cathrine Skov Schacksen, BSc (Aalborg University, Denmark)  Nanna Celina Henneberg, BSc (Aalborg University, Denmark)  Janusiya Anajan Muthulingam, MSc, PhD (Aalborg University, Denmark & Aalborg University Hospital, Denmark)  Yuh Morimoto, PhD (Juntendo University, Japan)  Ryuichi Sawa, PT, PhD (Juntendo University, Japan)  Masakazu Saitoh, MSc, PhD (Juntendo University, Japan)  Tomoyuki Morisawa, MSc, PhD (Juntendo University, Japan)  Nobuyuki Kagiyama, MD, PhD (Juntendo University, Japan)  Tetsuya Takahashi, PT, MSc, PhD (Juntendo University, Japan)  Takatoshi Kasai, MD, PhD (Juntendo University, Japan)  Hiroyuki Daida, MD, PhD (Juntendo University, Japan)  Jens Refsgaard, MD, PhD (Regional Hospital Viborg, Denmark)  Malene Hollingdal, MD, PhD (Regional Hospital Viborg, Denmark)  Birthe Dinesen, MSc, PhD (Aalborg University, Denmark) |
| Funding Sources |
| None |
| Conflicts of interest |
| Authors have no known conflicts of interest to declare |

| Review methods |
| --- |
| Review aim |
| This study reviews the literature on telerehabilitation within chronic heart failure (HF). The aim is to investigate the effects of telerehabilitation in the management of HF patients by conducting a scoping review of the available scientific literature within the period from January 1st 2015 to December 31st 2020. The effects on telerehabilitation that will be investigated are: Quality of life, physical capacity, depression, anxiety, and adherence of the intervention. |
| Literature Search |
| The literature search was conducted on the electronic databases PubMed and Excerpta Medica (EMBASE) by two of the review team members (CSS & NCH). The search protocol was developed using the guidelines for conducting scoping review provided by the Preferred Reporting Items for Systematic Reviews and Meta-analysis Extension for Scoping Reviews (PRISMA-ScR) checklist (http://www.prisma-statement.org/Extensions/ScopingReviews). |
| Participants/population |
| Participants diagnosed with HF who are older than 18 years. |
| Interventions/Exposures |
| Any home telerehabilitation interventions for patients with HF were included. The study needed to be a comparative study with telerehabilitation and traditional home care or other approaches. The study needed to have and report at least one of the following outcome measures: quality of life, physical capacity, depression/anxiety, or adherence of the intervention. |
| Types of study to be included initially |
| Randomized controlled trials, prospective intervention studies, reviews and meta-analysis were included. Protocols were excluded. Only English publications were included. |
| Search flow |

Two individual searches was conducted on Pubmed and EMBASE.

Search performed on PubMed January 2021 including search words and hits:

|  | Search words | Hits |
| --- | --- | --- |
| #1 | ((Heart failure[MeSH Terms]) OR (Heart failure[tiab])) OR (HF[tiab]) OR (telecardiology[tiab]) | 240,439 |
| #2 | (telerehabilitation[MeSH Terms]) OR (telerehabilitation[tiab]) | 1,232 |
| #3 | #1 AND #2 Filters: From 2015/01/01 to 2020/12/31 | **43** |

Search performed on EMBASE January 2021 including search words and hits:

|  | Search words | Hits |
| --- | --- | --- |
| #1 | 'heart failure'/exp OR 'heart failure' | 597,820 |
| #2 | 'telerehabilitation'/exp OR 'telerehabilitation' | 1,679 |
| #3 | #1 AND #2 Filters: From 2015/01/01 to 2020/12/31 | **67** |

| Data extraction and study records |
| --- |
| First, two review members (CSS and NCH) conducted the literature search, and duplicates were removed. The same two authors screened and evaluated titles and abstracts to whether it fulfilled the inclusion/exclusion criteria. Articles that fulfilled the inclusion/exclusion criteria were included in the review. Disagreements were resolved by discussion until consensus was reached. The following information was collected from all included studies: references, design of the study, sample size, severity of HF (reported as New York Heart Association Functional Classification (NYHA)), intervention type, technology, duration of intervention and follow-up, health care utilization, outcomes (QoL, physical capacity, depression, anxiety, adherence of the intervention), and questionnaires or tests used to measure QoL, physical capacity, depression and anxiety. An overview showing the main results relevant in the studies will be presented and only significant results will be reported in the overviews. Hence, we do not report tendencies. |
| Risk of bias assessment |
| Not applicable |

| Review general information |
| --- |
| Type of review |
| Scoping review |
| Language |
| English |
| Other registration details |
| Not applicable |
| Details of any existing review of the same topic by the same authors |
| Not applicable |
| Any additional information |
| Not applicable |
| Details of final report/publications |
| Will be submitted April 2021 |
